# The Innovative XClinic Tool: A Pilot Study Validating Its Precision in Measuring Range of Motion in Healthy Individuals

**DOI:** 10.3390/s25051331

**Published:** 2025-02-21

**Authors:** Giovanni Galeoto, Ilaria Ruotolo, Giovanni Sellitto, Emanuele Amadio, Enrica Di Sipio, Raffaele La Russa, Gianpietro Volonnino, Paola Frati

**Affiliations:** 1 Department of Human Neurosciences, Sapienza University of Rome, 00185 Rome, Italy; ilaria.ruotolo@uniroma1.it (I.R.); giovanni.sellitto@uniroma1.it (G.S.); emanueleama00@gmail.com (E.A.); 2 Department of Public Health and Infectious Diseases, Sapienza University of Rome, 00185 Rome, Italy; 3 IRCCS Neuromed, Via Atinense, 18, 86077 Pozzilli, Italy; 4 Euleria Health, Via delle Zigherane 4/A, 38068 Trento, Italy; enrica@euleria.it; 5 Department of Life, Health and Environmental Sciences, University of L’Aquila, 67010 L’Aquila, Italy; raffaele.larussa@univaq.it; 6 Department of Anatomical, Histological, Forensic and Orthopaedical Sciences, Sapienza University of Rome, 00185 Rome, Italy; gianpietro.volonnino@uniroma1.it (G.V.); paola.frati@uniroma1.it (P.F.)

**Keywords:** technology, inertial sensors, range of motion, XClinic, psychometric properties

## Abstract

Background: Kinematics experts and physical therapists have implemented the use of sensors for 3D motion analysis, both for static and dynamic movements. XClinic movement sensors are advanced devices designed to analyze movement patterns with high precision. The aim of this study was to validate wearable XClinic sensors for range of motion (ROM) in healthy subjects and obtain normative data. Participants were enrolled at the Sapienza University of Rome in 2024. All participants had to be healthy subjects aged between 18 and 65 years. Data on their demographics, employment and physical activity were collected. All the subjects were tested to assess the active ROM of their shoulder, hip, knee and ankle bilaterally. The same movements were tested using a goniometer to investigate validity, and SF-36 was administered. Fifty subjects were enrolled. The mean age was 28.2 (SD 10.8) years. For the left shoulder, construct validity showed statistically significant values for flexion, extension and extra-rotation, while for the right shoulder, construct validity showed statistically significant values for all movements except intra-rotation. The results concerning the right hip showed statistically significant values for flexion, extra-rotation, intra-rotation and adduction. The left hip showed statistically significant values for all movements except extension. Both the right and left knees showed statistically significant values for flexion. Both the right and left ankles showed statistically significant values for all movements. XClinic sensors offer a reliable and valid solution for the precise monitoring of the ROM of the shoulder and lower limb joints, making them an invaluable asset for clinicians and researchers.

## 1. Introduction

Rapid technological developments have occurred in recent years. Computer- and robot-assisted forms of therapy have proven to be an important component for optimizing rehabilitation [1,2]. The monitoring and analysis of bioelectrical signals and movements can aid in the diagnosis, prevention and examination of a wide range of issues. Remote monitoring and ambulatory monitoring are growing needs in the healthcare environment [3,4]. Objective and quantitative measurement is paramount in a detailed and reliable physical examination [5,6]. In recent years, biomedical engineers, kinematics experts and physical therapists have developed and implemented into research settings the use of quantitative sensors for 3D motion analysis, both for static and dynamic movements close to real life conditions described by patients and athletes [7,8]. To date, improvements in this field, including in the quality of data collection, a reduction in economic costs and user-friendly portable devices, have made these technologies increasingly widespread [9]. A real revolution started when kinematic sensors became wireless and wearable, allowing them to analyze ergonomics in the workplace or athletes’ performance on the field [10,11,12]. Among them, inertial sensors seem to be reliable, cost-effective and practical because they can integrate 3D data from a gyroscopic sensor, a magnetometer and an accelerometer to output a stable coordinate system expressed by Euler angles [13,14]. This new device should be tested to make the results equivalent to conventional measurement methods such as goniometry, dynamometry, optoelectronic kinematic analysis and ground contact force timing [15,16]. The possibility of using them as a certified alternative to common measurement methods in everyday clinical practice is interesting both for clinicians and patients, because with one device, a professional can collect more reliable data-saving time, money and space [17]. Furthermore, it is possible to have real-time feedback, which can be used for incorporating functional exercise prescriptions, telemedicine and objective outcome measures into rehabilitation programs and follow-ups [18]. XClinic movement sensors by Ferrox (Codognè, Italy) are advanced devices designed to monitor and analyze physical activities and movement patterns with high precision. In particular, they are able to measure an active range of motion of the shoulder, hip, knee and ankle and to assess balance in different positions. These sensors are particularly utilized in healthcare, sports and rehabilitation to provide detailed insights into a person’s motion. XClinic sensors are designed to provide precise and objective assessments of joint movement. These wearable and wireless devices are based on inertial sensors (accelerometer, gyroscope and magnetometer), allowing real-time monitoring of movement with high sensitivity. The key innovation of XClinic sensors lies in their ability to integrate and analyze data from multiple sensors, generating highly reliable measurements and reducing the margin of error typically associated with manual assessments, such as those performed with a traditional goniometer. Due to their compact and portable design, these sensors can be used not only in clinical and rehabilitation settings but also for sport performance monitoring and ergonomic applications, enabling a more detailed analysis of movement biomechanics. Additionally, the system’s digital interface allows for data storage and comparison over time, facilitating patient follow-ups and optimizing therapeutic strategies (https://www.ferrox.it/riabilitazione-digitale/, accessed on 20 February 2025). Overall, XClinic movement sensors by Ferrox provide an advanced solution for tracking and analyzing physical activity, delivering valuable insights that can significantly enhance health, performance and overall well-being [19].

For example, in post-surgical recovery (e.g., after knee or shoulder reconstruction), they can enable continuous tracking of ROM progress, ensuring that therapeutic interventions are optimized. In neurological rehabilitation, such as for patients with stroke-induced motor impairments, XClinic sensors can facilitate the precise monitoring of movement recovery and support tailored tele-rehabilitation programs. Moreover, in injury prevention, these sensors can detect subtle movement asymmetries that may predispose individuals to musculoskeletal injuries, allowing for early intervention strategies. XClinic sensors improve upon traditional methods like goniometry and optoelectronic motion capture by offering a portable, cost-effective and user-friendly solution. Unlike marker-based motion analysis, which requires specialized lab setups, XClinic sensors can be used in real-world settings—from rehabilitation clinics to sports training environments—without external calibration systems. Additionally, their real-time data processing and cloud-based integration enable automated movement analysis, potentially enhancing AI-driven rehabilitation protocols.

To further aid comprehension, incorporating conceptual diagrams illustrating sensor placement, data acquisition and clinical application scenarios would be beneficial. A comparison table between traditional ROM assessment methods and XClinic sensors could also highlight key advantages in terms of accuracy, portability and cost-effectiveness.

### Motivation

Recent advancements in biomechanical assessment and rehabilitation have highlighted the need for more precise, objective and user-friendly tools for monitoring joint mobility. Traditional methods, such as manual goniometry, often rely on the expertise of the clinician, leading to variability in measurements and requiring time-consuming procedures that are difficult to standardize across different settings. The XClinic system addresses these limitations by integrating wearable inertial sensors (accelerometers, gyroscopes and magnetometers), allowing for real-time, wireless and highly accurate range of motion (ROM) assessments. Unlike optical motion capture systems, which require laboratory setups, XClinic provides a portable and cost-effective alternative that can be used in clinical rehabilitation, sports performance monitoring and workplace ergonomics. In rehabilitation, it enables the continuous tracking of motor recovery in patients recovering from injuries or neurological conditions, optimizing treatment strategies. In sports, it allows for detailed movement analysis, helping athletes prevent injuries and enhance performance. Moreover, in occupational health, it facilitates postural and movement assessments to prevent work-related musculoskeletal disorders. By offering automated, reproducible and user-friendly motion analysis, XClinic enhances traditional biomechanical evaluations, making precise ROM tracking more accessible across multiple fields.

The aim of this study is to validate XClinic wearable sensors for ROM assessment in a sample of healthy subjects, to investigate their psychometric properties and obtain normative data on the devices.

## 2. Materials and Methods

This study was conducted by a research group at the Sapienza University of Rome named “Riabilitazione Evidenze e Sviluppo (RES)”, who were involved in different studies on rehabilitation [20,21,22].

### 2.1. Participants

The study was performed in accordance with the ethical standards as laid down in the 1975 Declaration of Helsinki and its later amendments. The participants of this cross-sectional study were enrolled at the Sapienza University of Rome from September 2023 to February 2024. Informed consent was obtained from all subjects involved in the study [23,24]. All participants had to be healthy subjects, with a Short Form Health Survey-36 (SF-36) score at least of 40% in the domain “General Health”; a Mini Mental State Examination (MMSE) score of over 24; and aged between 18 and 65 years. Exclusion criteria were road accidents and/or surgery for other reasons in the last year; neurological or cardio-pulmonary diseases; being pregnant and psychiatric conditions.

### 2.2. Data Collection

For each subject the following data were collected: demographics, Body Mass Index (BMI), employment and physical activity. All the subjects were tested to assess their active Range of Motion (ROM) relative to the following joints: shoulder, hip, knee and ankle bilaterally. For each joint, specific movements were tested:

- Shoulder: flexion, extension, abduction, internal rotation, external rotation.
- Hip: flexion (with flexed knee), extension, abduction, adduction, internal rotation, external rotation.
- Knee: flexion, extension.
- Ankle: plantar flexion, dorsiflexion, inversion, eversion.

The same movements were tested using a goniometer, the gold standard for the assessment of ROM, to investigate construct validity; also, muscle strength was tested using the Medical Research Council (MRC) scale, considering the following muscles, respectively, in the upper and lower limbs:

Upper limb muscles: anterior and posterior deltoid, pectoralis major, supraspinatus, subscapularis, infraspinatus, teres minor, biceps brachii and triceps brachii.

Lower limb muscles: iliopsoas, gluteus maximus, hamstring muscles, gluteus medius, gluteus minor, tensor fasciae latae, quadriceps, adductors, tibialis anterior, gastrocnemius, soleus, tibialis posterior, peroneus longus and peroneus brevis.

In addition to ROM and strength assessment, a Short Form Health Survey 36 (SF-36) questionnaire was administered to subjects [25].

The ROM assessment with both the goniometer and Xclinic sensors, the strength test and the administration of questionnaires were performed by three physiotherapists. Goniometer evaluation was performed following Hazel and Clarkson guidelines [26].

### 2.3. Evaluation Using Sensors

The Xclinic kit (Version 10) was composed as follows: two wearable sensors, charging equipment, adjustable straps and bands to secure the sensors in place on the body, instructional material and a user interface device (a tablet in this case) with the Xclinic app installed.

**Figure 1:**
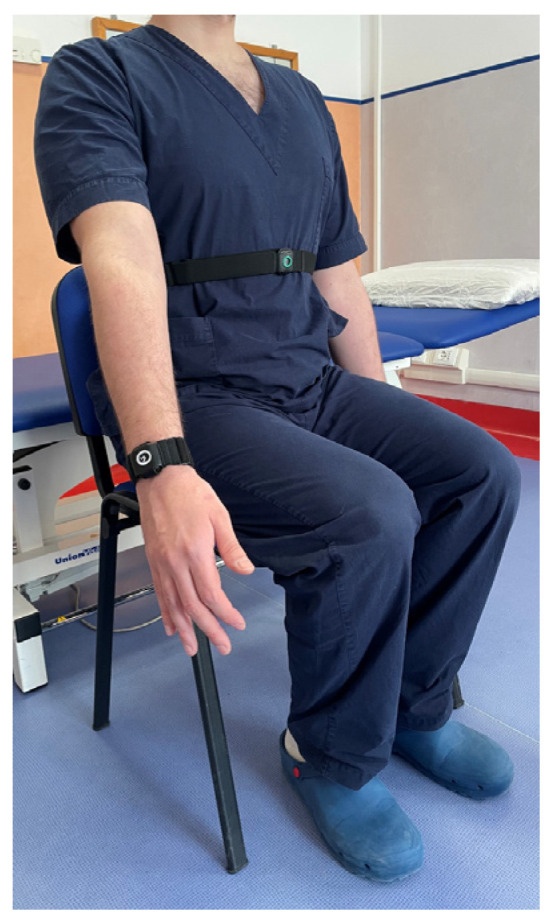
Sensor Placement—Shoulder.

**Figure 2:**
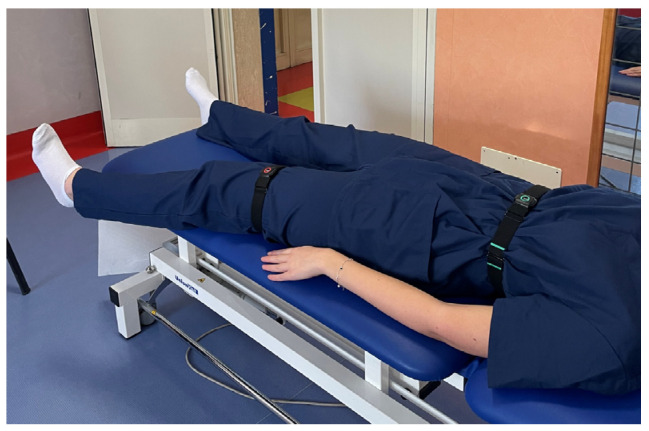
Sensor Placement—Hip.

**Figure 3:**
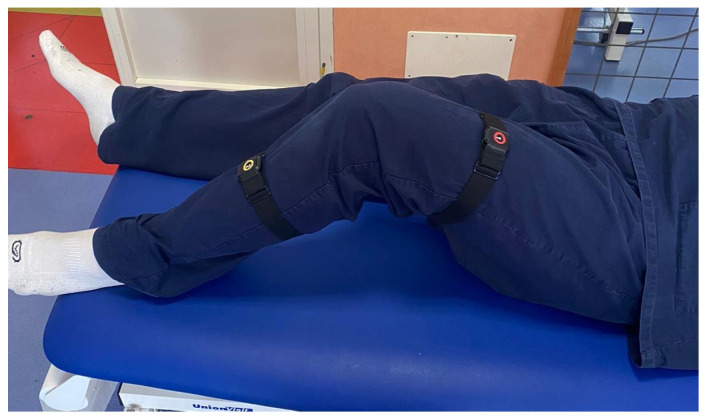
Sensor Placement—Knee.

**Figure 4:**
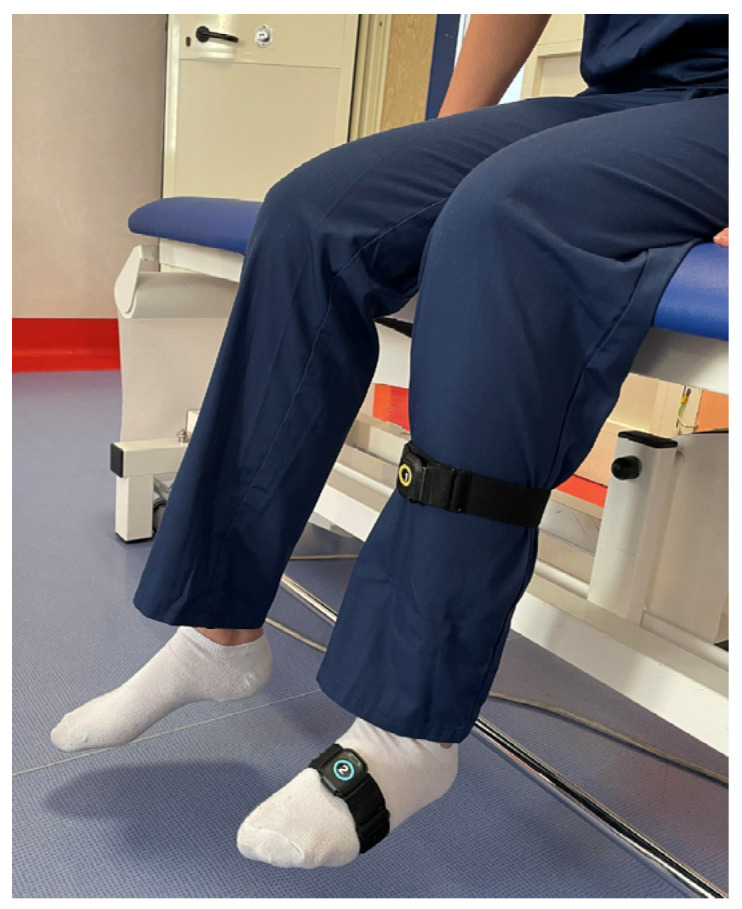
Sensor Placement—Ankle.

To effectively use the Xclinic sensors by Ferrox, the following steps were carried out:

- Unpacking and Initial Setup: sensors and related equipment were carefully unpacked and a check for any user manuals or quick start guides included in the package was performed.
- Powering the Sensors: before each evaluation, the operators ensured that the sensors were charged or connected to a power source.
- Connecting to a Network: specifically, to a local Wi-Fi network. The manufacturer’s instructions for connecting to the network were followed.
- Sensor Placement: sensors were placed in the locations to be monitored, precisely in correspondence with the upper and lower segment of the considered joint, to register the angle of movement. The operators ensured that the placement followed the guidelines provided by Ferrox. For each joint, the sensors were placed as follows: on the thorax and wrist for the shoulder (Figure 1), on the thorax and thigh for the hip (Figure 2), on the thigh and lower leg for the knee (Figure 3) and on the lower leg and foot for the ankle (Figure 4). This placement allowed the sensors to capture the relative motion between the segments, ensuring an accurate assessment of joint angles. Participants wore the Xclinic sensors during all movements.
- Configuration and Calibration: the software was used to configure and calibrate the sensors. The calibration process began by instructing the participant to remain immobile in a neutral anatomical position, allowing the software to establish baseline sensor offsets; a standardized setup and calibration process was followed.

Once a unique account was created for each subject in the Xclinic software (v. 1.0) and calibration was successfully completed, the assessment of range of motion (ROM) could begin. The software continuously recorded and stored movement data throughout the trials.

Participants followed a predefined movement protocol, which included specific instructions regarding range of motion and number of repetitions (3 for each movement). A trained researcher supervised each session to ensure correct execution and proper sensor function. Additionally, data collection was performed at a fixed sampling rate to ensure uniformity across participants. These procedures were implemented to guarantee the reliability and reproducibility of the measurements, minimizing any variability due to sensor misalignment or inconsistencies in movement execution.

The measurements using the Xclinic sensors and the goniometer were carried out separately, following a specific order: First, the range of motion (ROM) was assessed using the Xclinic sensors, and only afterward was the manual goniometer measurement performed. This sequence was chosen to ensure that the digital measurement remained unaffected by any prior manual positioning of the limb during the goniometric evaluation. To minimize variability between the two methods, several standardization procedures were followed. Participants were asked to perform the same predefined movements for both assessments. Additionally, the goniometric measurements were always taken by the same trained examiner to reduce any potential variability.

### 2.4. Statistical Analyses

The statistical analyses were performed using SPSS Statistics version 27. Demographic characteristics were reported as mean ± standard deviation (SD) or percentage, depending on the type of data. Internal consistency was assessed through item-by-item analyses for each movement, while construct validity was evaluated using Pearson’s correlation, comparing the ROM values obtained with the Xclinic sensors to those measured with a goniometer. Pearson’s correlation was also used to examine the relationship between ROM and SF-36 scores. The study followed the guidelines outlined in the “COnsensus-based Standards for the selection of health Measurement Instruments” (COSMIN) [27].

All data were initially entered into an Excel database, ensuring a structured and organized dataset before statistical analysis.

Once all measurements were collected, the raw data from Excel were imported into SPSS for processing. SPSS generated a new database where data procedures were applied to calculate psychometric properties. Descriptive statistics were then used to calculate the mean and standard deviation for each movement. A Pearson’s correlation analysis was performed to assess the relationship between the two measurement methods, as well as the association between ROM values and SF-36 scores. To evaluate reliability, internal consistency was analyzed through item-by-item correlations.

The final tables presented in Section 3 were derived from the SPSS database.

## 3. Results

### 3.1. Characteristics of the Sample

Fifty healthy subjects were enrolled. The mean age of the sample was 28.2, with a standard deviation of 10.8; 58% of the sample was composed by females. Among the included subjects 52% practiced sport, while 54% were workers and 76% were students. These results are shown in Table 1.

### 3.2. Reliability

#### Item-by-Item Analyses

The item-by-item evaluation of the left shoulder highlighted a statistically significant moderate direct correlation between flexion and extension, and intra and extra rotation. The results are shown in Table 2. The item-by-item evaluation of the right shoulder highlighted a moderate and statistically significant correlation between flexion and extension, extension and intra-rotation and intra and extra-rotation. The results for direct and inverse correlations are shown in Table 3.

As regards the item-by-item analyses of the left hip, the results show that there is a statistically significant inverse correlation between extension and extra rotation, adduction and abduction and extension and extra rotation. Right hip analyses show statistically significant direct correlations between intra and extra rotation, adduction and extra rotation, adduction and extra rotation, adduction and flexion, extension and extra rotation and flexion and extension. Data on direct and inverse correlations are shown in Table 4 and Table 5.

The item-by-item evaluation of the right knee highlighted a statistically significant direct correlation between flexion and extension ROM (Table 6 and Table 7).

The item-by-item evaluation of the right ankle showed a statistically significant correlation between the following movements: plantar and dorsal flexion, inversion and plantar flexion, eversion and dorsal flexion and eversion and inversion. Left ankle analyses highlighted a statistically significant correlation between dorsal and plantar flexion, eversion and dorsal flexion and inversion and dorsal flexion. The results for direct and inverse correlations are shown in Table 8 and Table 9.

Finally, item-by-item analyses was also performed with regards to shoulder ROM and SF 36 (Table 10). These highlighted a statistically significant correlation between left shoulder flexion and general health; shoulder extension and level of energy/fatigue; abduction and role limitations due to physical health; role limitations due to emotional problems, social functioning and pain and extra rotation and role limitations due to emotional problems and social functioning.

### 3.3. Construct Validity (Goniometric Evaluation)

Construct validity was carried out by comparing the value obtained through the sensors’ assessments with the goniometric values; the results concerning the left shoulder showed statistically significant values for flexion, extension and extra-rotation (moderate statistically significant correlations, *p* < 0.01). The results are reported in Table 11. The results concerning the right shoulder showed statistically significant values for all the movements except for intra-rotation (moderate statistically significant correlations, *p* < 0.01). The results are shown in Table 12 and Table 13.

The results concerning the right hip showed statistically significant values for flexion, extra-rotation, intra rotation and adduction. The results for the left hip showed statistically significant values for all the movements except for extension. Data are shown in Table 13 and Table 14.

The data regarding both the right and left knee showed statistically significant values for flexion ROM (Table 15 and Table 16).

The results regarding both the right and left ankle showed statistically significant values for all the assessed movements. Correlations are shown in Table 17 and Table 18.

### 3.4. Construct Validity (SF-36)

Construct validity was also performed by comparing the values obtained through sensors assessments with the scores obtained in several domains of SF-36 survey. Correlations are shown in Table 19, Table 20, Table 21 and Table 22.

## 4. Discussion

The objective of the study was to validate XClinic wearable sensors for ROM assessment in a sample of healthy subjects, and to investigate its psychometric properties. Item-by-item analyses were performed to evaluate the internal consistency of the Xclinic sensors. These analyses showed statistically significant correlations between specific movements; in particular, it is interesting to observe the inverse correlation found between flexion and extension in the shoulder joint. This result was also found between abduction and adduction in the hip joint, and in flexion and extension in the knee joint. An inverse correlation also exists between the following movements of the ankle: inversion and eversion, and plantar and dorsal flexion. These data suggest that Xclinic sensors are able to detect the difference between opposite movements in space; to date, no studies have investigated the internal consistency of measurements using inertial sensors.

This finding is consistent with the expected behavior of these joints, where an increase in one movement naturally corresponds to a decrease in the opposite movement. The fact that the Xclinic sensors detected these relationships suggests that they provide reliable and internally consistent measurements.

Secondly, the construct validity of the device was investigated through comparisons with goniometer measurements. The current literature is not extensive regarding studies focusing on the validation of inertial sensors vs. goniometers in measuring the ROM of joints. In our study, statistically significant values were found, in particular when comparing shoulder flexion and extension—in fact, Xclinic data relative to these movements were similar to those collected using a goniometer. This data is in line with the current literature, as stated in the study of Kaszyński et al. [28]. As regards both the hip and knee joint, the flexion ROM obtained the most significant values—that is to say, this represented the movement that most correlated with the values measured with the goniometer. In general, rotation movements show less agreement with those measured with the goniometer; this resulted in these movements being more complicated to measure, because the equipment required more accurate placing, and this suggests the necessity of training professionals to deal with Xclinic sensors. This data on rotation assessments is also concordant with the current literature [29]. The joint that showed more statistically significant correlations between goniometer and Xclinic evaluation was the ankle, with regards to all the studied movements (plantar and dorsal flexion, inversion, eversion); the reason for this could be that these are smaller movements, so they are easier to perform and to assess with both the device and goniometer. To date, as far as we know, no studies have investigated the validity of inertial sensors specifically in the ankle joint for healthy individuals.

However, the study presents some limitations: Firstly, the sample is composed of only 50 subjects; it would be recommended to increase the number to obtain more significant data. In addition, the goniometer is considered a standard tool for assessing the ROM of any joint in the physiotherapeutic or orthopedic field, but it has a significant limitation: the obtained results often depend on the professional’s skills and experience [30]. Recommendations for future studies should concern a more detailed investigation of the tool’s psychometric properties, such as its reliability, to prove its reproducibility over time, as well as its responsiveness, to demonstrate its capacity to detect changes in specific clinical conditions.

## 5. Medico-Legal Implications

From a medico-legal perspective, this technological aid—aimed at the analysis of joint ROM—can find different fields of application. First of all, according to Law 219/2017, it is always important to respect the patient’s health and to act in total safety, thus providing all the necessary information about the treatment and its purposes. In addition, the correct assessment of joint functional limitations and postural analysis is the fulcrum of patient assessment in the field of social security medicine. Given the great sensitivity to the issue of disability, an objective and scientifically valid assessment provides an excellent aid for patients who need social benefits. Finally, it should be remembered that, in terms of civil liability, victims of road accidents are entitled to compensation, paid on the basis of the biological damage caused, which will be quantified on the basis of the clinical and instrumental data. In this case too, a meticulous damage evaluation, supported by specific markers, makes it possible to provide an accurate estimate of the functional limitation—allowing on the one hand the formulation of the right diagnosis and the correct therapeutic process, and also allowing the patient to obtain adequate compensation.

## 6. Conclusions

The evaluation of the Xclinic sensors by Ferrox underscores their substantial validity and reliability in clinical and environmental monitoring applications. Through rigorous testing and comparison with the established gold standard, these sensors have demonstrated a good degree of accuracy and consistency in data collection. Their robust design, ease of use, and advanced features ensure that they meet the demands of modern healthcare and environmental monitoring, providing professionals with dependable and precise data.

In summary, Xclinic sensors by Ferrox offer a reliable and valid solution for precise monitoring of the ROM of shoulder and lower limb joints, making them an invaluable asset for clinicians and environmental scientists alike. Their accuracy and consistency proven here can now reinforce their status as a leading choice in sensor technology, promoting better decision making and improved outcomes in their respective fields. Finally, this approach can have a great impact at the medico-legal level, both in terms of ethics and in the context of correct biological damage assessment.

## Figures and Tables

**Table 1:** Characteristics of the sample.

	Average ± SD	N
Age	28.2 ± 10.8	50
		(N%)
Age Ranges	20–24	22 (44)
	25–29	18 (36)
	30–64	10 (20)
Gender	Female	29 (58)
Sport		26 (52)
Workers		27 (54)
Students		38 (76)

**Table 2:** Item-by-item correlation for left shoulder.

	Flexion	Extension	Abduction	Intrarotation	Extrarotation
Flexion	1	−0.405 **	0.169	−0.021	0.301 *
Extension	−0.405 **	1	−0.209	−0.194	−0.255
Abduction	0.169	−0.209	1	0.237	0.226
Intrarotation	−0.021	−0.194	0.237	1	0.299 *
Extrarotation	0.301 *	−0.255	0.226	0.299 *	1

* *p* < 0.05; ** *p* < 0.01.

**Table 3:** Item-by-item correlation for right shoulder.

	Flexion	Extension	Abduction	Intrarotation	Extrarotation
Flexion	1	−0.292 *	0.182	0.186	0.175
Extension	−0.292 *	1	0.073	−0.300 *	−0.109
Abduction	0.182	0.073	1	−0.064	0.151
Intrarotation	0.186	−0.300 *	−0.064	1	0.418 **
Extrarotation	0.175	−0.109	0.151	0.418 **	1

* *p* < 0.05; ** *p* < 0.01.

**Table 4:** Item-by-item correlation for left hip.

	Extrarotation	Intrarotation	Adduction	Abduction	Flexion	Extension
Extrarotation	1	0.238	0.035	−0.019	−0.080	−0.335 *
Intrarotation	0.238	1	0.054	−0.246	0.184	−0.037
Adduction	0.035	0.054	1	−0.290 *	0.045	−0.016
Abduction	−0.019	−0.246	−0.290 *	1	0.183	0.244
Flexion	−0.080	0.184	0.045	0.183	1	0.266
Extension	−0.335 *	−0.037	−0.016	0.244	0.266	1

* *p* < 0.05; ** *p* < 0.01.

**Table 5:** Item-by-item correlation for right hip.

	Extrarotation	Intrarotation	Adduction	Abduction	Flexion	Extension
Extrarotation	1	0.663 **	0.349 *	0.042	0.055	−0.386 **
Intrarotation	0.663 **	1	0.024	0.048	0.191	−0.254
Adduction	0.349 *	0.024	1	−0.262	0.295 *	−0.117
Abduction	0.042	0.048	−0.262	1	−0.145	−0.087
Flexion	0.055	0.191	0.295 *	−0.145	1	0.286 *
Extension	−0.386 **	−0.254	−0.117	−0.087	0.286 *	1

* *p* < 0.05; ** *p* < 0.01.

**Table 6:** Item-by-item correlation for left knee.

	Flexion	Extension
Flexion	1	0.152
Extension	0.152	1

* *p* < 0.05; ** *p* < 0.01.

**Table 7:** Item-by-item correlation for right knee.

	Flexion	Extension
Flexion	1	0.821 **
Extension	0.821 **	1

* *p* < 0.05; ** *p* < 0.01.

**Table 8:** Item-by-item correlation for left ankle.

	Plantar Flexion	Dorsal Flexion	Eversion	Inversion
Plantar flexion	1	−0.469 **	−0.028	0.371 **
Dorsal flexion	−0.469 **	1	0.542 **	0.171
Eversion	−0.028	0.542 **	1	0.328 *
Inversion	0.371 **	0.171	0.328 *	1

* *p* < 0.05; ** *p* < 0.01.

**Table 9:** Item-by-item correlation for right ankle.

	Plantar Flexion	Dorsal Flexion	Eversion	Inversion
Plantar flexion	1	−0.526 **	−0.001	0.105
Dorsal flexion	−0.526 **	1	0.406 **	0.353 *
Eversion	−0.001	0.406 **	1	0.194
Inversion	0.105	0.353 *	0.194	1

* *p* < 0.05; ** *p* < 0.01.

**Table 10:** Item-by-item correlation between shoulder and SF-36.

		Physical Functioning	Role Limitations Due to Physical Health	Role Limitations Due to Emotional Problems	Energy/ Fatigue	Emotional Well-Being	Social Functioning	Pain	General Health
Left	Flexion	0.181	−0.112	0.058	0.062	0.204	0.037	0.038	0.382 **
	Extension	0.015	−0.068	−0.061	0.384 **	0.181	0.037	−0.112	−0.058
	Abduction	0.084	0.472 **	0.505 **	−0.037	0.149	0.361 *	0.359 *	0.072
	Intrarotation	−0.146	0.025	0.094	−0.176	−0.080	−0.003	−0.079	−0.062
	Extrarotation	0.154	0.179	0.280 *	0.172	0.284 *	0.323 *	0.217	0.234
Right	Flexion	0.021	0.017	0.086	−0.111	0.034	0.019	0.026	−0.080
	Extension	−0.193	−0.175	−0.256	0.211	0.116	−0.042	−0.277	−0.030
	Abduction	0.032	−0.011	0.004	−0.048	−0.023	0.066	0.011	0.033
	Intrarotation	−0.054	0.211	0.186	−0.174	0.067	0.157	0.090	−0.079
	Extrarotation	0.027	0.084	0.279	0.055	0.145	0.269	0.073	−0.027

* *p* < 0.05; ** *p* < 0.01.

**Table 11:** Left shoulder construct validity.

		Goniometric Evaluation				
		Flexion	Extension	Abduction	Intrarotation	Extrarotation
Xclinic evaluation	Flexion	0.532 **	−0.382 **	−0.115	0.044	0.338 *
	Extension	0.045	0.554 **	0.083	0.163	−0.132
	Abduction	0.061	−0.103	0.075	−0.105	0.162
	Intrarotation	−0.291 *	−0.036	−0.154	0.244	−0.007
	Extrarotation	0.002	−0.083	0.097	0.292 *	0.589 **

* *p* < 0.05; ** *p* < 0.01

**Table 12:** Right shoulder construct validity.

		Goniometric Evaluation				
		Flexion	Extension	Abduction	Intrarotation	Extrarotation
Xclinic evaluation	Flexion	0.575 **	−0.339 *	0.004	0.088	0.204
	Extension	−0.284 *	0.559 **	−0.240	0.065	−0.078
	Abduction	−0.055	−0.299 *	0.015	−0.157	0.218
	Intrarotation	0.133	−0.060	−0.220	0.477 **	0.241
	Extrarotation	−0.011	−0.030	−0.193	0.221	0.612 **

* *p* < 0.05; ** *p* < 0.01

**Table 13:** Right hip construct validity.

		Goniometric Evaluation					
		Extrarotation	Intrarotation	Adduction	Abduction	Flexion	Extension
Xclinic evaluation	Extrarotation	0.559 **	0.441 **	0.200	−0.096	0.129	−0.220
	Intrarotation	0.393 **	0.744 **	0.096	−0.087	0.291 *	−0.098
	Adduction	0.200	0.065	0.537 **	−0.003	0.215	−0.132
	Abduction	0.078	0.113	0.206	0.170	−0.130	−0.026
	Flexion	0.185	0.397 **	0.303 *	0.164	0.771 **	0.015
	Extension	0.130	0.137	−0.152	0.440 **	−0.015	0.393 **

* *p* < 0.05; ** *p* < 0.01

**Table 14:** Left hip construct validity.

		Goniometric Evaluation					
		Extrarotation	Intrarotation	Adduction	Abduction	Flexion	Extension
Xclinic evaluation	Extrarotation	0.344 *	0.028	0.223	−0.166	0.151	0.167
	Intrarotation	0.284 *	0.763 **	0.151	−0.116	0.258	−0.025
	Adduction	0.203	0.055	0.292 *	−0.115	0.010	−0.085
	Abduction	0.153	−0.016	0.154	0.530 **	0.075	0.135
	Flexion	0.300 *	0.298 *	0.120	0.295 *	0.677 **	0.168
	Extension	0.239	0.154	−0.171	0.385 **	−0.052	0.265

* *p* < 0.05; ** *p* < 0.01

**Table 15:** Right knee construct validity.

		Goniometric Evaluation	
		Flexion	Extension
Xclinic evaluation	Flexion	0.702 **	0.027
	Extension	0.136	0.213

* *p* < 0.05; ** *p* < 0.01.

**Table 16:** Left knee construct validity.

		Goniometric Evaluation	
		Flexion	Extension
Xclinic evaluation	Flexion	0.724 **	−0.001
	Extension	0.054	0.265

* *p* < 0.05; ** *p* < 0.01.

**Table 17:** Right ankle construct validity.

		Goniometric Evaluation			
		Plantar Flexion	Dorsal Flexion	Eversion	Inversion
Xclinic evaluation	Plantar flexion	0.653 **	−0.395 **	0.114	0.283 *
	Dorsal flexion	−0.179	0.604 **	0.300 *	0.190
	Eversion	0.104	0.126	0.535 **	0.179
	Inversion	0.293 *	−0.020	0.208	0.722 **

* *p* < 0.05; ** *p* < 0.01.

**Table 18:** Left ankle construct validity.

		Goniometric Evaluation			
		Plantar Flexion	Dorsal Flexion	Eversion	Inversion
Xclinic evaluation	Plantar flexion	0.741 **	−0.537 **	−0.077	0.364 **
	Dorsal flexion	−0.323 *	0.558 **	0.417 **	0.041
	Eversion	−0.072	0.368 **	0.778 **	0.372 **
	Inversion	0.285 *	−0.017	0.186	0.693 **

* *p* < 0.05; ** *p* < 0.01

**Table 19:** Shoulder construct validity (SF-36).

		Physical Functioning	Role Limitations Due to Physical Health	Role Limitations Due to Emotional Problems	Energy/ Fatigue	Emotional Well-Being	Social Functioning	Pain	General Health
Left	Flexion	0.181	−0.112	0.058	0.062	0.204	0.037	0.038	0.382 **
	Extension	0.015	−0.068	−0.061	0.384 **	0.181	0.037	−0.112	−0.058
	Abduction	0.084	0.472 **	0.505 **	−0.037	0.149	0.361 *	0.359 *	0.072
	Intrarotation	−0.146	0.025	0.094	−0.176	−0.080	−0.003	−0.079	−0.062
	Extrarotation	0.154	0.179	0.280 *	0.172	0.284 *	0.323 *	0.217	0.234
Right	Flexion	0.021	0.017	0.086	−0.111	0.034	0.019	0.026	−0.080
	Extension	−0.193	−0.175	−0.256	0.211	0.116	−0.042	−0.277	−0.030
	Abduction	0.032	−0.011	0.004	−0.048	−0.023	0.066	0.011	0.033
	Intrarotation	−0.054	0.211	0.186	−0.174	0.067	0.157	0.090	−0.079
	Extrarotation	0.027	0.084	0.279	0.055	0.145	0.269	0.073	−0.027

* *p* < 0.05; ** *p* < 0.01.

**Table 20:** Hip construct validity (SF-36).

		Physical Functioning	Role Limitations Due to Physical Health	Role Limitations Due to Emotional Problems	Energy/ Fatigue	Emotional Well-Being	Social Functioning	Pain	General Health
Left	Extrarotation	−0.357 *	0.019	0.086	−0.129	−0.130	0.028	−0.195	−0.272
	Intrarotation	−0.099	−0.045	−0.013	0.006	0.034	−0.004	0.071	0.077
	Adduction	−0.093	−0.027	0.041	0.056	0.213	0.085	−0.028	0.156
	Abduction	0.011	−0.059	−0.119	0.151	0.062	0.030	−0.071	−0.005
	Flexion	0.025	0.029	−0.044	0.303 *	0.372 **	0.135	0.176	0.306 *
	Extension	0.214	0.019	0.161	0.208	0.198	0.165	0.170	0.009
Right	Extrarotation	−0.240	0.090	0.060	0.027	−0.007	0.055	−0.039	−0.161
	Intrarotation	−0.124	−0.005	0.023	0.148	0.049	−0.007	0.034	0.013
	Adduction	−0.073	−0.128	−0.290 *	−0.032	0.130	−0.073	−0.139	0.060
	Abduction	0.029	0.136	0.124	0.200	0.214	0.181	0.161	0.335 *
	Flexion	0.079	0.007	−0.009	0.228	0.298 *	0.095	0.116	0.301 *
	Extension	0.291 *	0.101	0.185	0.057	0.199	0.193	0.216	0.017

* *p* < 0.05; ** *p* < 0.01.

**Table 21:** Knee construct validity (SF-36).

		Physical Functioning	Role Limitations Due to Physical Health	Role Limitations Due to Emotional Problems	Energy/ Fatigue	Emotional Well-Being	Social Functioning	Pain	General Health
Left	Flexion	0.189	0.052	0.123	0.056	0.189	0.123	0.228	0.130
	Extension	0.082	0.214	0.287 *	0.239	0.351 *	0.357 *	0.206	0.218
Right	Flexion	0.187	−0.007	−0.019	−0.004	0.168	0.041	0.054	0.156
	Extension	0.171	0.037	0.102	0.076	0.213	0.157	0.188	0.191

* *p* < 0.05; ** *p* < 0.01.

**Table 22:** Ankle construct validity (SF-36).

		Physical Functioning	Role Limitations Due to Physical Health	Role Limitations Due to Emotional Problems	Energy/ Fatigue	Emotional Well-Being	Social Functioning	Pain	General Health	Lefs-I
Left	Plantar flexion	−0.009	0.046	−0.113	−0.092	0.005	−0.039	0.076	0.161	0.000
	Dorsal flexion	0.122	0.025	−0.021	0.079	0.055	0.007	0.026	−0.119	0.222
	Eversion	0.023	−0.097	−0.190	−0.053	0.018	−0.195	−0.057	−0.084	0.233
	Inversion	0.201	0.091	0.013	−0.175	0.015	0.002	0.166	−0.085	0.226
Right	Plantar flexion	0.033	−0.021	−0.191	−0.272	−0.211	−0.255	0.057	−0.227	0.197
	Dorsal flexion	0.017	−0.014	0.077	0.218	0.147	0.081	−0.006	0.062	0.103
	Eversion	−0.239	−0.033	−0.125	−0.202	−0.091	−0.153	−0.142	−0.192	0.038
	Inversion	0.225	0.033	0.133	0.007	0.217	0.094	0.129	0.133	0.332 *

* *p* < 0.05; ** *p* < 0.01.

## Data Availability

The raw data supporting the conclusions of this article will be made available by the authors on request.
